# Prospective Assessment of No Evidence of Disease Activity-4 Status in Early Disease Stages of Multiple Sclerosis in Routine Clinical Practice

**DOI:** 10.3389/fneur.2019.00788

**Published:** 2019-07-24

**Authors:** Carlos Guevara, Cristian Garrido, Melissa Martinez, Gonzalo A. Farias, Patricia Orellana, Wendy Soruco, Pablo Alarcón, Violeta Diaz, Carlos Silva, Matthew J. Kempton, Gareth Barker, José de Grazia

**Affiliations:** ^1^Faculty of Medicine, Hospital Clínico José Joaquín Aguirre, Santos Dummont, Universidad de Chile, Santiago, Chile; ^2^Institute of Psychiatry, Psychology & Neuroscience, King's College London, London, United Kingdom

**Keywords:** relapsing/remitting, quantitative MRI, outcome measurement (NEDA-4), multiple sclerosis, brain atrophy

## Abstract

**Background:** In relapsing-remitting multiple sclerosis, no evidence of disease activity-3 (NEDA-3) is defined as no relapses, no disability progression and no MRI activity. NEDA-4 status is defined as meeting all NEDA-3 criteria plus having an annualized brain volume loss (a-BVL) of ≤0.4%. Prospective real-world studies presenting data on NEDA-4 are scarce.

**Objective:** To determine the proportion of patients failing to meet one or more NEDA-4 criteria and the contribution of each component to this failure.

**Methods:** Forty-eight patients were followed for 12 months. Structural image evaluation, using normalization, of atrophy was used to assess a-BVL.

**Results:** The patients had a mean age of 33.0 years (range 18–57), disease duration of 1.7 years (0.4–4) and Expanded Disability Status Scale score of 1.3 (0–4); 71% were women. All patients were on disease-modifying therapies. During follow-up, 21% of the patients had at least one relapse, 21% had disability progression, 8% had new T2 lesions, and 10% had gadolinium-enhanced lesions. Fifty-eight percent (28/48) achieved NEDA-3 status. a-BVL of >0.4% was observed in 52% (25/48). Only 29% (14/48) achieved NEDA-4 status.

**Conclusion:** a-BVL is a good marker to detect subclinical disease activity. a-BVL is parameter to continue investigating for guiding clinical practice in relapsing-remitting multiple sclerosis.

## Introduction

The characterization of disease progression in relapsing-remitting multiple sclerosis (RRMS) to test the effectiveness of disease-modifying therapies (DMTs) is needed. Identifying subclinical signs of disease activity is imperative to prevent the inflammatory and neurodegenerative aspects of RRMS and reduce progression to irreversible disability.

Combined disease status assessments are increasingly explored to evaluate the overall impact of DMTs (1). No evidence of disease activity-3 (NEDA-3) is defined as the absence of all of the following: relapses, disability progression, and MRI activity (new/enlarged T2 lesions and/or gadolinium-enhanced T1 lesions). However, individual components of NEDA-3 may be somewhat impractical and underused in routine clinical practice for assessing ongoing disease activity. In a large retrospective cohort (1,594 patients with RRMS), 810 patients showed evidence of disease activity (EDA) (≥1 relapse or an increase in the Expanded Disability Status Scale (EDSS) score by ≥0.5 points and/or MRI activity) after at least 2 years of follow-up (2). Of these 810 patients, 31.9% were assessed as having progressive disease and 64.8% as stable; despite the clinical and MRI changes, the treating neurologist did not recommend treatment optimization in the putatively stable patients. Thus, the criteria for NEDA-3 may not be suitable for the determination of timely treatment failure in routine clinical practice.

Disease activity follow-up may be improved by considering quantitative magnetic resonance imaging (qMRI) as soon as RRMS is diagnosed. In the present study, structural image evaluation, using normalization, of atrophy (SIENA) (3, 4) was used to explore brain volume loss (BVL) in RRMS and to investigate the addition to NEDA-3 of a fourth criterion—no pathological BVL—proposed by Kappos et al. (5) During normal aging, the annual rate of cerebral BVL has been estimated as ≤0.4% (6, 7). NEDA-4 status is therefore defined as meeting all NEDA-3 criteria plus having an annualized BVL (a-BVL) of ≤0.4% (5).

Accelerated a-BVL is predictive of disability progression and cognitive decline in the long term (8, 9). NEDA-4 outcomes have almost exclusively been reported from randomized controlled trials (RCTs) in carefully selected groups of patients;(5) prospective real-world studies presenting data on NEDA-4 are scarce.

We aim to determine the proportion of patients failing to meet one or more NEDA-4 criteria and the contribution of each component to this failure in RRMS patients with early disease stages in the routine clinical practice.

## Materials and Methods

### Patient Assessment

Forty-eight adult patients with RRMS according to the revised McDonald criteria (10) were followed at the University of Chile Hospital. This observational study was made as part of the routine clinical practice and therefore the treating neurologists were free to made any therapeutic change. Between January 2016 and March 2018, patients underwent two clinical assessments and two MRI brain scans. The second clinical assessment and MRI brain scan were performed approximately 12 months after the baseline assessment. For both the baseline and follow-up assessments, the clinical data and MRI scans were acquired within 1 week of each other. Patients were first time assessed when no relapse and at least 90 days after the last relapse. All patients were on DMT (Table 1). Due to the observational nature of the study, no further inclusion criteria were defined; however, patients were excluded if they had a clinical disease duration of ≥4 years and an EDSS score of ≥4. Patients underwent clinical and MRI assessments at screening and at month 12. The following definitions were used for the individual components of NEDA-3 (1): relapse: the appearance of a new or the worsening of a previously stable neurological abnormality, present for at least 24 h and occurring in the absence of fever or infection, confirmed within 7 days of symptom onset; focal MRI activity: new or enlarged T2 lesions and/or gadolinium-enhanced T1 lesions; and confirmed disability progression: an increase in the EDSS score of at least 0.5 points from the baseline score. Neuropsychological status was assessed using the Brief International Cognitive Assessment for Multiple Sclerosis (BICAMS) and scores were assessed relative to normative data in a Hispanic population (11, 12). At 12 months, worsening of at least 10% of the BICAMS measures and of the three instruments that compose BICAMS (the Symbol Digit Modalities Test (SDMT), California Verbal Learning Test 2 (CVLT2) and the Brief Visual Spatial Memory Test-Revised (BVMTR) were considered to be clinically meaningful after one year of follow-up (11, 12).

**Table 1:** Baseline demographic and baseline and follow-up disease characteristics.

**Characteristics**	***n***: 48	
Baseline age, years, mean (SD)	33.0 (10.5)	
Female, *n* (%)	34 (71%)	
Mean disease duration of RRMS since diagnosis (SD), years	1.7 (1.4)	
Expanded Disability Status Scale score, mean (range), baseline and follow-up	1.3 (0–4)	1.3 (0–4.5)
Brief International Cognitive Assessment for Multiple Sclerosis, mean (SD), baseline and follow-up	121 (25)	125 (29)
Symbol Digit Modalities Test	43.5 (13)	45.3 (12)
California Verbal Learning Test 2	54.3 (10)	54.4 (12)
Brief Visual Spatial Memory Test-Revised	23.9 (7)	25.3 (7)
Disease-modifying therapy, *n* (%), baseline and follow-up		
Interferon	22 (44)	13 (27)
Fingolimod	16 (34)	23 (44)
Glatiramer acetate	8 (15)	8 (17)
Teriflunomide	2 (4)	2 (4)
Natalizumab	0	1 (2)
Alemtuzumab	0	1 (2)
Relapses before 1^st^ visit, *n* (%) one	31 (65%)	
two or more	17 (35%)	
T2 lesions, *n* (%), baseline and follow-up. <10	16 (33%)	7 (15%)
11–50	30 (60%)	27 (56%)
>50	2 (6.4%)	12 (25%)
Gadolinium-enhanced T1 lesions, *n* (%), baseline and follow-up.	9 (19%)	5 (10%)
Proportion of patients who failed to meet NEDA-4 criteria		
Relapses	21% (10/48)	
EDSS progression	21% (10/48)	
New T2 lesions	8% (4/48)	
Gadolinium-enhanced T1 lesions	10% (5/48)	
a-BVL >0.4%	52% (25/48)	
Proportion of patients achieving NEDA-3 status	58% (28/48)	
Proportion of patients achieving NEDA-4 status	29% (14/48)	

*RRMS, relapsing-remitting multiple sclerosis; EDSS, Expanded Disability Status Scale; a-BVL, annualized brain volume loss; NEDA, no evidence of disease activity*.

### MRI Acquisition

For both the baseline and follow-up assessments, the clinical data and MRI scans were acquired within 1 week of each other. Brain MRI was performed in baseline and follow-up on the same MRI system using the same imaging protocol (that is, the same pulse sequences and spatial resolution). MRI images were acquired on a 1.5 T Siemens MRI Scan. Axial T1-weighted images of the whole brain were obtained using a 3D inversion-recovery prepared spoiled gradient-echo (IR-SPGR) sequence. The following parameters were used in this volumetric sequence: field of view of 250 × 234 mm; matrix of 256 × 240 mm; repetition time of 12 ms; echo time of 5.68 ms; excitation flip angle of 15°; isotropic voxel size of 0.98 × 0.98 × 0.98 mm. Patients underwent a second MRI brain scan at the time of the final study visit [12 months after the baseline scan]. Two neuroradiologists (JdG and PO, one of them with 20 years experience and the other with 5 years experience) assessed the MRI scans of every patient to rule out gross anatomical abnormalities. No MRI images included in this study showed any structural abnormalities other than atrophy-related changes and demyelinating lesions. New lesions in follow up (T2 lesions and gadolinium-enhancing lesions) were defined as any new area more than 3 mm hyperintense on T2 / FLAIR or with gadolinium enhancement on T1, evaluated by eye. The enlargement of T2 lesions from baseline to follow-up was also evaluated by eye. No quantitative measure was made regarding T2 lesion load.

The number of T2 lesions per patient scan was divided into three categories: <10; 11–49; and more than 50 (13).

### Data Analysis

All images were converted to NIFTI format using MRIcron software (http://people.cas.sc.edu/rorden/mricron/dcm2nii.html). Cross-sectional whole brain volumes and brain tissue volumes were estimated using SIENAX (3). Before further processing, all data were anonymized by removing any reference to the patients' names from the image headers. Briefly, SIENAX extracts brain and skull images from the acquired MRI data. The brain image is then affine registered to Montreal Neurological Institute 152 space, using the skull image to determine the registration scaling. The registration scaling is then used to obtain a volumetric scaling factor, which is employed to normalize the tissue volume estimates. Segmentation with partial volume estimation is subsequently performed to calculate the total volume of brain tissue, including separate estimates of the volumes of gray matter (GM) and white matter (WM). The gray matter is divided into cortical gray matter (cGM) and deep gray matter (dGM) (14). The longitudinal SIENA processing algorithm has been validated and described in detail elsewhere (3). The processing steps are as follows: (1) Brain extraction (BET): segmentation of brain from non-brain tissue for each scan, followed by skull extraction. (2) Registration: registration of the segmented brain from the second (follow-up) scan to that of the first (baseline) scan using a linear transformation. The two skull images are used as normalizing factors to constrain the scale and skew. (3) Tissue type segmentation: white matter and gray matter tissues are treated as one tissue and the cerebrospinal fluid as another tissue. (4) Change analysis: detection of the edge of the brain on the registered baseline and follow-up image. At each edge point the displacement between the baseline brain edge and follow-up brain edge is determined. Finally, the mean displacement of brain surface at each edge point is converted to a global percentage change in brain volume by taking into account the baseline brain volume.

Subjects were included in the study if they had two MRI scans of adequate quality and the brain extraction step in SIENA functioned correctly.

SIENA is freely available (http://www.fmrib.ox.ac.uk/analysis/research/siena).

### Statistical Analyses

Statistical analyses of the clinical data were performed using Statistical Package for Social Sciences, version 22. The results are presented as the means ± SDs. In all cases, a two-sided p of <0.05 was considered significant. Comparisons between groups were assessed using the *t*-test. a-BVL was calculated by dividing the BVL values by the interscan interval in years. Clinical scores were annualized by dividing the unit change between the assessments by the assessment interval in years.

**Figure 1:**
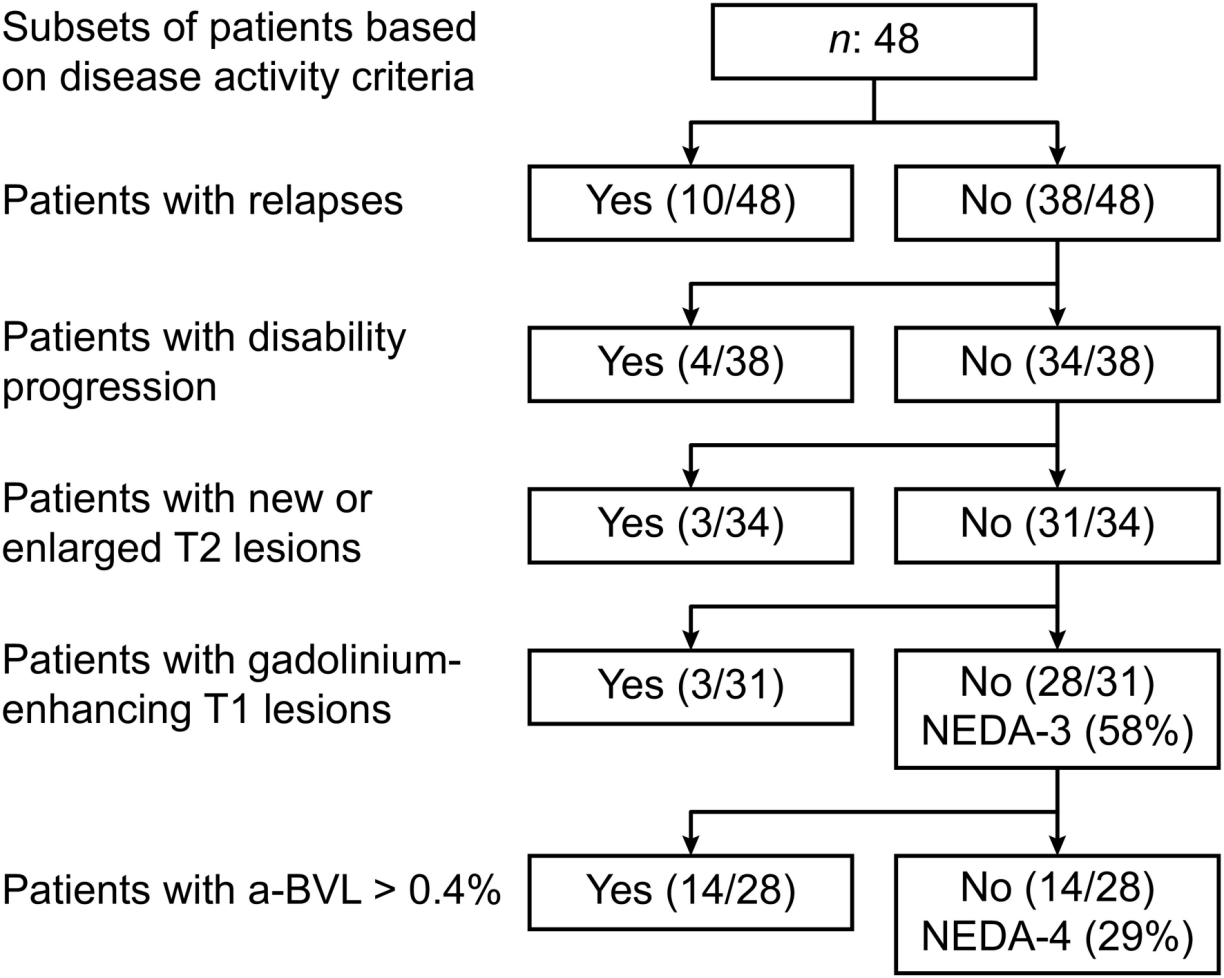
Hierarchical analysis of individual disease activity criteria.

To assess the contribution of the four different components of the NEDA-4 measure, hierarchical analysis of patients was performed according to individual disease activity criteria using the following hierarchy: relapses, disability progression, MRI activity and accelerated a-BVL. In the analysis, patients who had an event for one outcome were removed from evaluation for any other outcomes from that point on. For example, if a patient had a relapse, the patient was removed from subsequent evaluation for disability progression, MRI activity and a-BVL (Figure 1).

### Standard Protocol Approval and Patient Consent

Prior to inclusion, patients provided informed written consent for participation in the study. The study was conducted in accordance with international standards of good clinical practice (ICH guidelines and the Declaration of Helsinki). The project was approved by the local research ethics committees of the University of Chile Hospital, Santiago, Chile.

## Results

### Baseline Assessment (Table 1)

The mean age of the patients was 33.0 years (range 18–57), 71.0% were women, the mean disease duration was 1.7 years (0.4–4), and the mean EDSS score was 1.3 (0–4). Fifty-two percent of the patients performed poorly on the overall BICAMS. None of the patients had other neurological diseases affecting the central nervous system or had a history of alcohol/substance dependence or abuse. Before the first visit, 65.0% (31/48) of the patients had experienced one relapse and 35.0% (17/48) had experienced two or more relapses. The DMTs were interferon (44.0%), fingolimod (34.0%), glatiramer acetate (15.0%) and teriflunomide (6.0%).

Thirty-three percent of the patients had less than 10 T2 lesions, 60.6% had between 11 and 49 lesions, and 6.4% had more than 50 lesions. Gadolinium-enhanced T1 lesions were observed in 19.0% of the patients.

In the current study, segmentation of brain from non-brain tissue did not fail for each scan, additional pre-processing steps were not needed, and every pair of images was included in the analysis. All qMRI volumes were within normal limits: mean whole brain, 1,562 ml (range 1,344–1,660); gray matter, 748 ml (642–802); peripheral gray matter, 582 ml (502–643); and white matter, 814 ml (702–893) (Table 2).

**Table 2:** Quantitative MRI volumetric data in RRMS: normalized brain tissue volume and annualized brain volume change.

	**Baseline**	**Follow-up**	***p*-value^c^**
Annualized brain volume loss, mean% ± SD (range)^a^		0.56%, ±SD 0.79; (3.13 to −0.65)^b^	
Normalized brain volume, mL, ± SD mean (range)	1562 ± 60 (1,344–1,660)	1551 ± 64 (1,303–1,650)	0.031
Peripheral gray matter, mL, mean (range) ± SD	582 (502–643) 31	576 (437–652) 39	0.049
Gray matter, mL, mean (range) ± SD	748 (642–802) 35	741 (625–807) 39	0.028
White matter, mL, mean (range) ± SD	814 (702–893) 38	810 (677–879) 39	0.118
Gray matter loss and brain volume loss correlation			*p* <0.001 *r*: 0.6

a *The mean scan interval was 1.02 ± 0.05 years. a-BVL was calculated by dividing the BVL values by the interscan interval in years*.

b Negative values imply a volume increase. Positive values imply brain volume loss (BVL).

c *Comparisons between groups were assessed using the t-test*.

### Follow-Up Assessment

A total of 21.0% (10/48) of the patients had at least one relapse, 21.0% (10/48) had disability progression, 8.0% (4/48) had new/enlarged T2 lesions, and 10.0% (5/48) had gadolinium-enhanced T1 lesions. A total of 58% of the patients (28/48) achieved NEDA-3 status. An a-BVL of >0.4% was observed in 52.0% (25/48) [mean: 0.56%, ±SD 0.79; range 3.13 to −0.65 (negative values imply a volume increase)]. Changes in the a-BVL were driven by changes in the total gray matter (*p*: 0.028) and cGM (*p*: 0.049) (Table 2). The white matter volume did not change significantly (*p*: 0.118). With the addition of the a-BVL criterion, 29.0% (14/48) achieved NEDA-4 status.

BICAMS declined by 10% in 3 patients (6%), the Symbol Digit Modalities Test deteriorated in 8 patients (17%), California Verbal Learning Test in 10 (21%) and the Brief Visual spatial Memory Test-Revised declined in 10 (21%). BICAMS correlated negatively well with the EDSS score (*r*: −0.37; *p* = 0.01) and age (*r*: −0.50; *p* < 0.001). In the hierarchical analysis, when adding BICAMS 50% of patients would achieved a putative NEDA-4.

During the course of the follow-up, nine patients were assessed as having active disease by the treating neurologists. Treatment switching occurred in nine patients (19%) from interferons to fingolimod (n:7), alemtuzumab (n:1), and natalizumab (n:1).

The only baseline factor that differed between patients with NEDA-4 and patients with EDA was the number of relapses before the first assessment (*p*: 0.034). A total of 29% (14/48) of the patients had accelerated a-BVL only, without a deterioration in any other component of NEDA-4 (Table 1 and Figure 1).

## Discussion

The frequency of conversion from RRMs to a secondary progressive multiple sclerosis (SPMS) increases with duration of disease (12% at 5 years; 41% at 10 years) (15). Assessing BVL early during the course of the disease could help identify groups of people with RRMS that may benefit from particular types of therapies based on their stage of disease and before the progression to SPMS, when patients seem to receive no benefit from DMTs. In the words of Tofts, qMRI should ideally be able to express in an “easy, reproducible, comparable, and convenient way the deviation from normality of some MRI parameters of the brain tissue early in the course of the disease” (16). Assessing BVL from qMRI data is an informative and unbiased way to quantify disease progression and tissue loss.

The current work includes a-BVL values collected in a prospective assessment of NEDA-4 with disease duration and disease disability constraints in the context of clinical practice rather than in the experimental setting of RCTs. This longitudinal study shows a mean a-BVL change of 0.56% ± SD 0.79. This rate is higher than the mean reported for healthy individuals (0.1–0.3%) (8) and falls within the range of annual rates reported in a pivotal RCT in RRMS using the same analysis method (SIENA) (17).

The pathological processes responsible for atrophy are likely to involve the death of different types of brain cells. In the cohort reported here, cGM and dGM losses were the contributors to accelerated a-BVL. Gray matter loss and brain volume loss were highly correlated (*r*: 0.6 and *p* < 0.001). However, the number of patients who had gray matter loss >0.4% were 45% vs. patients who had BVL >0.4%: 52%. We feel that the small sample and these figures do not allow us to propose gray matter loss as a putative component in NEDA algorithms.

In contrast to supratentorial white matter volume, which did not significantly change after 1 year of follow-up. These *in vivo* data provide further evidence that tissue loss in the cGM and dGM structures occurs early in the course of RRMS. Indeed, many longitudinal studies have shown that gray matter atrophy is a better predictor of disease progression than white matter atrophy (18, 19). A recent large multicenter longitudinal study in 3604 patients augmented this finding by showing that that dGM loss drives disability accumulation in RRMS (20). However, the accurate segmentation of GM is difficult to achieve. The cortex (cGM) is a thin layer of GM surrounded by WM on one side and CSF on the other, both of which produce partial volume effects that confound its delineation. Moreover, the automated segmentation of dGM is much less accurate than that of cGM. For instance, the automated techniques tended to misclassify large portions of dGM as WM (21). These shortcomings in the segmentation of GM structures should be taken into account when interpreting these findings.

The components of NEDA-3 may reflect the ongoing disease status imperfectly, and their variability limits their effectiveness as outcome measures. Relapses and gadolinium-enhanced lesions on T1-weighted scans may reflect only focal inflammatory disease activity, underestimating the presence of early diffuse and clinically silent neurodegeneration in RRMS (22).

New/enlarging lesions on T2-weighted scans are one of the main parameters used for following disease activity in RRMS. However, in clinical practice, the detection of new or enlarged T2 lesions is limited by technical and methodological factors. Manual counting of T2 lesions is imprecise, and the number of new T2 lesions is typically specified only approximately or as >10 when many are present (13). Moreover, the cortical lesion burden is poorly visualized by routine MRI protocols (19). In RCTs, T2 lesion volumes are often automatically counted, which may more accurately assess of the T2 lesion burden (17). In the current study, the longitudinal measurement of T2 lesions was a rather insensitive parameter for assessing disease progression, as the T2 lesion burden accounted for only 6% (3/48, Figure 1) of the unique events.

We also show that in our clinical cohort, failure to achieve NEDA-4 status was mainly driven by accelerated a-BVL. Baseline disease activity may help predict whether patients will achieve NEDA-4 status. In the present study, when patients were stratified by baseline disease activity, the number of relapses before the first assessment was significantly lower in patients who achieved NEDA-4 status than in those who did not. However, the baseline characteristics that affected NEDA-4 status in the present study differed from those in a retrospective multicenter real-world study (23), which reported that the proportion of patients (mean disease duration of 8 years, median age of 42 years) achieving NEDA-4 status was greater among those with lower EDSS scores and fewer gadolinium-enhanced T1 lesions; these differences may reflect differences in patient populations between these studies, particularly regarding disease duration.

The results of this study suggest that gray matter changes underlie early cognitive impairment in RRMS. Depicting precise cognitive profiles in patients with RRMS would thus potentially assist therapeutic decisions, especially at earlier stages. However, cognitive impairment tools deserve further evaluation as key factors in therapeutic algorithms for RRMS. Sacca et al. (24) has integrated BICAMS and two orientation tests (Mini Mental State Examination and the Montreal Cognitive Assessment) instead of the Cerebral Functional System into the EDSS scoring. They have shown that the sensitivity to detect cognitive impairment in a cross-sectional fashion increased by 25% in the group of patients with EDSS score <4. In the current cohort, cognitive impairment was already present at baseline in at least 50% of these rather young patients (25). At the group level, this percentage did not increase in the following year and thus BICAMS may have not been a sensitive marker of disease progression when there is a floor effect with high proportion of cognitive impairment at baseline. However, cognitive impairment may be a key domain to consider when choosing the first line therapy.

This study does have limitations, however. Although the a-BVL value has been widely used as an outcome in clinical trials, the critical question becomes whether the treating neurologists would consider switching therapy to potentially more effective drugs in patients who have not achieved NEDA-4 status. This topic is controversial, particularly regarding patients who have accelerated a-BVL only (29% in this study). Although SIENA has been shown to have a low estimation error for atrophy rate over the whole brain (0.5%) (3, 4, 14). confounding factors in determining the rate of BVL require further discussion. The follow-up period in the present study seems to be clinically meaningful for switching the initial DMT owing to disease activity, but the 1-year period may overestimate BVL because of the resolution of the early anti-inflammatory effect of DMTs and steroids (pseudoatrophy). Thus, a 2-year period has been suggested as a more robust approach when measuring BVL (26). However, in individual patients, in the setting of potential pseudoatrophy, acute inflammation would be evidenced clinically with a deterioration in any component of NEDA-3, particularly focal MRI activity and relapses, and BVL would need to be assessed considering this caveat. BVL assessment at 6, 12, and 24 months may be a more appropriate accurate approach for assessing the pattern of disease activity and overcoming the confounding factor of pseudoatrophy.

In addition, concerns regarding the biological validity of these BVL changes in RRMS remain. Indeed, the standard deviation 0.79% exceeded by far the cut-off of BVL and the broad range of change suggests a great of data variability. Factor such as alcohol (27), mild traumatic brain injury (28), smoking, genetics, diabetes mellitus (29), and hydration/dehydration can cause changes in brain size (30). Moreover brain volumes seem to fluctuate throughout the day, decreasing from morning to evening (31). Although these clinical factors may add to the variance in the measurements at the individual level, making it more difficult to detect real changes, clinicians, and patients may be able to allow for these potential confounders.

Various sources of error related to image acquisition can affect MRI atrophy quantification: image artifacts due to head motion, poor signal-to-noise ratio, partial head coverage, imperfect patient repositioning in a longuitudinal study and image adquisition with non-identical scan parameters. Even small changes could be argued to be an artifact caused by, for example, cardiac pulsations. However, due to the duration of the MRI acquisition, all images are effectively an average over several minutes, so the effects of cardiac pulsation should be averaged out.

We have not included the spinal cord assessment related to the lesions and atrophy, this limitation is of clinical significance as spinal cord pathology is a major contributor to RRMS disability. Indeed, the rate of spinal cord atrophy is greater than that of brain atrophy (1.78% vs. 0.5% per year) (32, 33) suggesting that spinal cord atrophy is a sensitive and meaningful marker of neurodegeneration (32). Spinal cord atrophy-related measures are calculated using semi-automated segmentation-based methods, which are subject to inter-rater variability. Future directions of research to fully automated analysis methods, including segmentation of gray matter and intramedullary lesions will facilitate the use of spinal cord atrophy in the clinical and research arenas (33).

A strength of this study is the prospectively collected clinical data, with a high quality control standard that supports the feasibility of assessing NEDA-4 status in clinical practice. Throughout the duration of the study, the patients underwent the same MRI protocol on the same MRI scanner at a single site. When the patients are prospectively recruited in a single center the risks of data variability may be substantially reduced.

The current diagnostic criteria and the follow-up tools of disease progression in RRMS lack any relevance to the neurodegenerative aspects and concentrate mainly on the inflammatory process. BVL may be a cornestone of measurement neurodegenerative components of disease progression RRMS, which should lead to improvement in treatment strategies and patients outcomes. However, current methods provide sufficient precision for cohort studies, but are not adequate for confidently assessing changes in individual patients. Advancing in imaging and processing techniques will enable neurologists to probe BVL along with the clinical endpoints in RRMS and ultimately improve treatment (34).

## Conclusion

Substantial evidence indicates that uncontrolled clinical and subclinical disease activity in early stages of RRMS may be critical for the evolution of long-term disability. The sequential addition of the individual components of NEDA-4 results in fewer patients achieving NEDA status at 1 year of follow-up. Brain atrophy is a good marker of disease progression in RRMS, and a-BVL is a parameter to continue investigating for guiding clinical practice.

## Ethics Statement

The study was conducted according to International Standards of Good Clinical Practice (ICH guidelines and the Declaration of Helsinki). The project was approved by the local Research Ethics Committees of Universidad de Chile Hospital, Santiago, Chile.

## Author Contributions

CGu: conception, organization, execution, review, and critique. CGa, MM, GF, PO, WS, PA, VD, CS, MK, GB, and JdG: review and critique.

### Conflict of Interest Statement

GB receives honoraria for teaching from General Electric Healthcare. The remaining authors declare that the research was conducted in the absence of any other commercial or financial relationships that could be construed as a potential conflict of interest.
